# International Medical Congress

**Published:** 1887-09

**Authors:** 


### *INTERNATIONAL MEDICAL CONGRESS.*

The preliminary work in all the departments of the Congress is progressing very satisfactorily. From this city there will be a great number in attendance. Dr. Judson B. Andrews, who is president of the Psychological Section, will read a paper on "The Distribution and care of the Insane in the United States." Dr. Floyd S. Crego, who is a member of the Council of the Psychological Section, will read on the "Cause and Cure of Sleeplessness" before his section; he will also take part in the discussion of the subject, "The Relation of Syphilis to Insanity." The work of this section has progressed most favorably, as we would expect under the management of a gentleman with such executive ability as Dr. Andrews. The non-participation of the New York and Philadelphia physicians is to be regretted, but the time of the section is so fully taken up by noted men from abroad, that it would be difficult to see how any space could have been given to them had they joined the section. That all will be present there is no doubt. Dr. Geo. E. Fell will give a description of his already-celebrated and successful effort to resuscitate a person with opium narcosis by means of laryngotomy and the bellows.

It is said, for well-known reason, that in several sections the eminent men in this country have failed to respond to the call to participate, but the talent from all the foreign countries is of the very highest. The work will commence promptly on Monday, Sept. 5th.

The following, from the *American Medical Journal*, will prove of interest:

CONDITIONS OF MEMBERSHIP IN THE NINTH INTERNATIONAL MEDICAL CONGRESS.

"Rule 1. The Congress will consist of such members of the regular medical profession as shall have registered and taken out their ticket of admission, and of such other scientific men as the Executive Committee of the Congress may deem desirable to admit. The dues of membership for residents of the United States will be ten dollars ($10). There will be no dues for members residing in other countries. Each member will be entitled to receive a copy of the *Transactions* of the Congress when published by the Executive Committee.

This rule, plainly defining the conditions for acquiring membership and participation in the approaching International Medical Congress, was adopted and published in English,

French and German, in Circular No. 1, issued and widely distributed in both this country and Europe by the Executive Committee more than two years ago. It was repeated in Circular No. 2, issued in July, 1886, and also republished in most of the medical periodicals of this country. And yet we notice that some of our State and local medical societies have appointed *delegates* to the Congress, and we are often receiving letters making inquiries touching the same subject. Hence, we have again quoted the rule, and wish to state explicitly that the doors of the International Medical Congress will be thrown open to all members of the regular medical profession, in all countries where such profession exists, who may apply to the Registration Committee in Washington, D. C., enter th ir names in full on the roll, and take their tickets of admission. Those residing in this country must pay, at the time of registering, the $10. From those residing in other countries no fee will be required.

In regard to the registration of educated dentists, about which there has been some question, it is sufficient to say that the same rule will be followed as governed at the London Congress of 1881. The establishment of a Section of Oral and Dental Surgery is a full admission that it constitutes a part of the domain of general medicine and surgery, and that all who, by education and proper legal authority, practice in that special department, are "members of the regular medical profession." At the London Congress they registered with the common prefix "Dr.," as did a large proportion of eminent members of the profession in other departments. At the Congress in Washington it will be proper for them to register with the title Dr., M. D., D. M. D., or D. D. S., according to the terms of the authority conferring upon them the right to practice their profession."

The fares on all the railroads have been reduced to one and one-third full fare for the round trip for members and their families, but all who wish to avail themselves of the reduced rates must apply to J. W. H. Lovejoy, M. D., No. 900 Twelfth street, Washington, D. C., for just as many certificates as they have persons intending to go.

